# Identification and functional characterization of the first molluscan neuromedin U receptor in the slug, *Deroceras reticulatum*

**DOI:** 10.1038/s41598-020-79047-x

**Published:** 2020-12-18

**Authors:** Seung-Joon Ahn, Rory J. Mc Donnell, Jacob A. Corcoran, Ruth C. Martin, Man-Yeon Choi

**Affiliations:** 1 Horticultural Crops Research Unit, USDA-ARS, Corvallis, OR USA; 2 Department of Biochemistry, Molecular Biology, Entomology & Plant Pathology, Mississippi State University, Mississippi State, MS USA; 3 Department of Crop and Soil Science, Oregon State University, Corvallis, OR USA; 4 Biological Control of Insects Research Unit, USDA-ARS, Columbia, MO USA; 5 Forage Seed and Cereal Research Unit, USDA-ARS, Corvallis, OR USA

**Keywords:** Entomology, Peptides, G protein-coupled receptors

## Abstract

Neuromedin U (NmU) is a neuropeptide regulating diverse physiological processes. The insect homologs of vertebrate NmU are categorized as PRXamide family peptides due to their conserved C-terminal end. However, NmU homologs have been elusive in Mollusca, the second largest phylum in the animal kingdom. Here we report the first molluscan NmU/PRXamide receptor from the slug, *Deroceras reticulatum*. Two splicing variants of the receptor gene were functionally expressed and tested for binding with ten endogenous peptides from the slug and some insect PRXamide and vertebrate NmU peptides. Three heptapeptides (QPPLPRYa, QPPVPRYa and AVPRPRIa) triggered significant activation of the receptors, suggesting that they are true ligands for the NmU/PRXamide receptor in the slug. Synthetic peptides with structural modifications at different amino acid positions provided important insights on the core moiety of the active peptides. One receptor variant always exhibited higher binding activity than the other variant. The NmU-encoding genes were highly expressed in the slug brain, while the receptor gene was expressed at lower levels in general with relatively higher expression levels in both the brain and foot. Injection of the bioactive peptides into slugs triggered defensive behavior such as copious mucus secretion and a range of other anomalous behaviors including immobilization, suggesting their role in important physiological functions.

## Introduction

Neuromedin U (NmU) is a neuropeptide, identified initially from the porcine spinal cord^1^. Since then, NmU has been found in many vertebrates and shown to regulate diverse physiological processes including smooth-muscle contraction, blood pressure, local blood flow, ion transport in the gut, stress responses, gastric acid secretion and feeding behavior in vertebrates^2,3^. All NmU peptides isolated from vertebrates have a highly conserved C-terminal sequence (-PRNamide), which is closely related to the C-terminal sequence (-PRXamide, X indicates a variable amino acid) of insect PRXamide family peptides and invertebrate orthologs of NmU^4^.

Several mammalian NmU receptors (NmU-Rs) have been deorphanized and characterized in the gut and central nervous system (CNS) and found to be involved in feeding behavior^5–8^. Genetic screening of the *Drosophila* genome found that G protein-coupled receptors (GPCRs) (CG8784, CG8795, CG9918, and CG14575) are homologs of the vertebrate NmU-Rs^9,10^. These GPCRs have been identified as receptors for insect PRXamide family peptides, including pyrokinins (PKs) and CAPA peptides^11–14^. Upon the peptides binding to their corresponding GPCRs, G-proteins coupled are activated to open a ligand-gated calcium channel to allow the influx of extracellular Ca^2+^ as a second messenger, triggering a cascade of signal transduction for essential cellular processes^10,15^. These neuropeptides are involved in feeding behavior, muscle contraction, and fluid secretion in the digestive organs^16–19^. Four NmU-R homologs of the nematode *Caenorhabditis elegans* have also been identified as receptors for a nematode PRXamide^20^.

Mollusca, which includes snails and slugs, is the second largest phylum in terms of species diversity in the animal kingdom, and together with Arthropoda, Annelida, and Nematoda makes up the Protostomia clade. From an evolutionary viewpoint, therefore, the molluscan components are crucial to infer invertebrate evolution. The first Molluscan NmU/PRXamide family peptides were identified in the terrestrial snail, *Helix lucorum*^21^. However, no molluscan receptors for NmU/PRXamide family peptides have been characterized yet, although a variety of neuropeptides and physiological signals have been extensively studied in sea slugs in the genus *Aplysia*. The slug species, *Deroceras reticulatum* (Müller) (Gastropoda: Pulmonata), is a terrestrial slug native to Europe with global distribution^22^ that causes serious damage in a wide range of vegetables and field crops^23^. Unlike shelled snails, the terrestrial slugs including *D. reticulatum*, are highly sensitive to desiccation, sometimes showing a huddling behavior with a contracted posture when humidity is low^24,25^. Recently, we mined the slug transcriptome to develop a comprehensive list of neuropeptide genes including those that encode neuropeptides with a PRXamide at the C-termini^26^.

In this study, we identified two splicing variants of a slug receptor gene that respond to the NmU/PRXamide peptides from *D. reticulatum*. The receptors were heterologously expressed and tested with a variety of natural and synthetic peptide ligands, not only deorphanizing the receptors, but also characterizing the essential peptide moieties for receptor binding. This is the first report on the molluscan NmU/PRXamide receptor and its true ligands. It sheds light on the molecular evolution of the signaling system between vertebrate NmU and invertebrate PRXamide neuropeptides in animals.

## Results

### Identification of six genes encoding NmU-like peptides in slugs

Our previous study identified seven different genes encoding putative NmU/PRXamide peptides in the *D. reticulatum* transcriptome^26^. In the current study, their full-length genes were cloned by PCR, sequenced, and named *myomodulin1*, *myomodulin2* and *myomodulin3*; *pleurin1* and *pleurin2*; and *small cardioaccelery peptides* (*sCAP1* and *sCAP2*) (GenBank Accession numbers: MW026240–MW026246). Twenty-six NmU/PRX peptides were predicted from the deduced amino acid sequences (Table 1, Fig. S1). The *myomodulin1* gene produces 16 myomodulin-like peptides, including eight canonical Mmd peptides with a conserved MLRLamide(a) at their C-terminal ends (Table 1, Fig. S1). The PMSMLRLa was the most abundant with 7 out of 16 copies, and there were two copies for each of the QLSMLRLa and SLGMLRLa peptides. The *myomodulin2* gene produces two myomodulin peptides, including a GQFSAARLa, a GLQMLRLa, and three NmU/PRX peptides, QPPLPRYa, FFFRPAPRGa, and AVPRPRIa. The *myomodulin3* gene produces four NmU/PRXamide peptides, including two copies of QPPVPRYa, and single copies of the QPPLPRYa and SFFRPAPRGa peptides. The NmU/PRXamide peptides from the *myomodulin2* and *myomodulin3* genes have the same or very similar amino acid sequences.

**Table 1:** NmU/PRXamide peptides predicted from the precursor genes in *Deroceras reticulatum*.

Gene	Peptide family	Sequence	Copy
*Myomodulin1*	Myomodulin	GGYDMLRLa	1
		GLNMLRLa	1
		KMSMLRLa	1
		QLSMLRLa	2
		SLGMLRLa	2
		ALGMLRLa	1
		AMSMLRLa	1
		PMSMLRLa	7
*Myomodulin2*	Myomodulin	GQFSAARLa	1
		GLQMLRLa	1
	NmU/PRX	QPPL**PRY**a	1
		FFFRPA**PRG**a	1
		AVPR**PRI**a	1
*Myomodulin3*	NmU/PRX	QPPV**PRY**a	2
		QPPL**PRY**a	1
		SFFRPA**PRG**a	1
*Pleurin1*	NmU/PRX	VFYTKSDDNDY**PRI**a	1
		SFYTRGSDTHY**PRI**a	1
		GIFTQSAHGSY**PRV**a	1
*Pleurin2*	NmU/PRX	VFFTKASDNDY**PRI**a	1
		SNFFTSGNGNHY**PRI**a	1
		GVFTQGPHGSY**PRV**a	1
*sCAP1*	NmU/PRX	MQYLAF**PRM**a	1
		SGYLAF**PRM**a	1
*sCAP2*	NmU/PRX	LNYLAF**PRM**a	1
		SGYLAF**PRM**a	1

The conserved C-terminal motif (-PRXamide) in NmU/PRXamide peptides is indicated with bold letters. The full-length prepropeptide sequences are found in the supplementary Figure S1.

The *pleurin1* and *pleurin2* genes produce six NmU/PRXamide peptides in total (Table 1, Fig. S1). They are composed of 14–15 amino acids and include four -PRIa peptides and two -PRVa peptides based on their C-terminal ends. The *sCAP1* and *sCAP2* genes produce four NmU/PRXamide peptides composed of nine amino acids, all with -PRMa C-terminal ends (Table 1, Figure S1). We selected eight representative peptides for the receptor-binding assays below.

### Identification of the NmU receptor genes in slug

**Figure 1:**
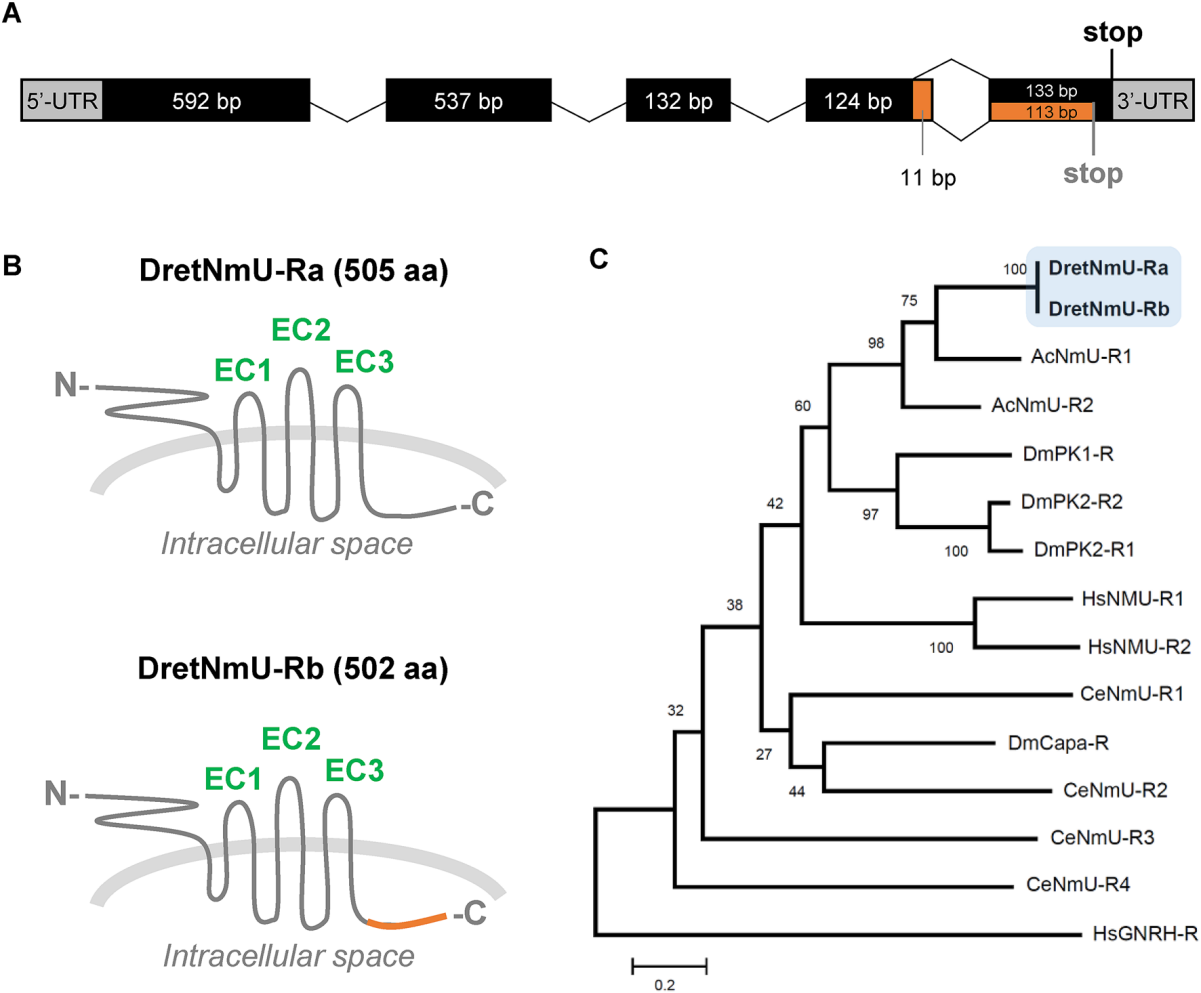
Structure of NmU receptor from *Deroceras reticulatum*. (**A**) Gene structure of the receptor composed of five exons, where two splicing variants (DretNmU-Ra and DreNmU-Rb) are derived. (**B**) Scheme of the two receptor variants, depicting the variable regions at the C-terminal intracellular domains. EC, extracellular (EC) domains. (**C**) Maximum-likelihood phylogenetic tree of the NmU receptor sequences from *D. reticulatum* (Dret), *Aplysia californica* (Ac), *Drosophila melanogaster* (Dm), *Caenorhabditis elegans* (Ce), and *Homo sapiens* (Hs). A human gonadotropin-releasing hormone receptor (GNRH-R) was used as an outgroup.

A BLAST search against the *D. reticulatum* transcriptome database was used to detect a 412-bp long transcript (c9058), which showed the highest homology to the vertebrate NmU receptors or the arthropod PRX receptors (E-value < 1e−59). RACE-PCR with gene-specific primers was used to amplify both ends of the gene, including 5′- and 3′-untranslated regions (UTRs). The full-length gene was then amplified from cDNA using conventional PCR and sequenced. Sequencing revealed two PCR amplicons representing two transcript variants, named DretNmU-Ra and DretNmU-Rb (GenBank Accession numbers: MW026247 and MW026248) (Fig. 1). When aligned with the slug genome sequences (*unpublished data*), the coding region of the receptor gene was found to contain five exons spanning about 9 Kb in the genome (Fig. 1A).

A comparison with the genome sequence revealed that the two transcript variants, -Ra and -Rb, seemed to be produced by alternative splicing events in exon 4. The *DretNmU-Rb* has 11 more nucleotides in exon 4 but 20 fewer nucleotides in exon 5 than DretNmU-Ra (Fig. 1A). A large intron gap (2250 bp) was found between exons 4 and 5 by PCR amplification using the specific primers for the two exons and genomic DNA. We confirmed the 11-bp retained in the variant -Rb and the resulting shift in the amino acid coding frame. Overall, *DretNmU-Rb* encodes a protein 3 aa-shorter (502 aa) than the variant -Ra (505 aa) (Fig. 1B, Supplementary data, Fig. S3). Protein sequence alignment by Clustal W algorithm and transmembrane (TM) domain prediction by TMHMM Server (v. 2.0, http://www.cbs.dtu.dk/services/TMHMM/) showed that the variable portion of these proteins is in the intracellular C-terminal domain, which is beyond the predicted seventh TM domain (Figs. 1B, S2). The phylogenetic tree of the NmU and PRX receptors indicated that the two slug receptors are close to *Drosophila* PK receptors and human NmU receptors (Fig. 1C).

### Functional testing of receptors

#### Screening with peptide ligands

**Figure 2:**
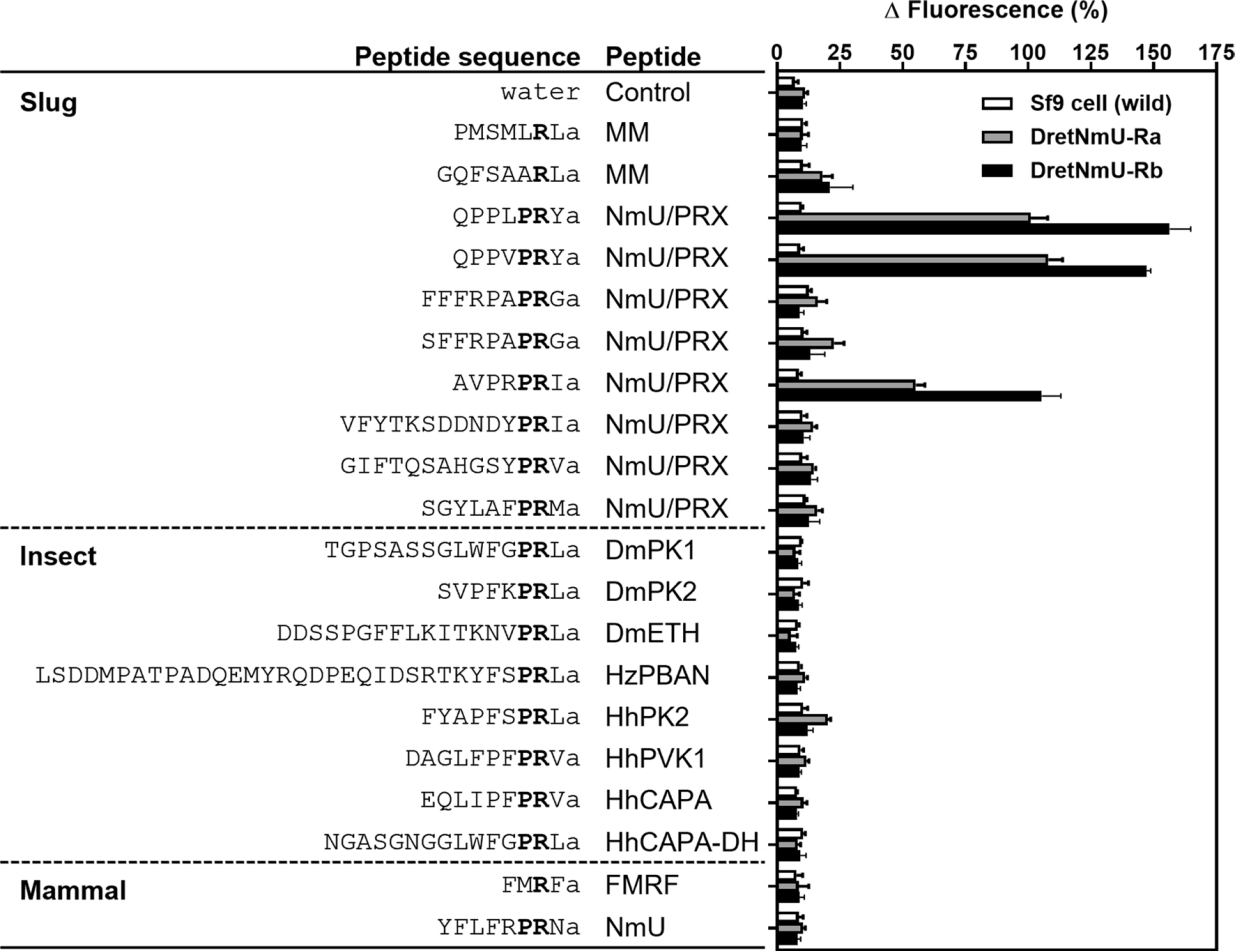
Ligand screening of the binding activity of two receptor variants identified from *Deroceras reticulatum*. Peptides derived from various sources including the slug were assayed at 500 nM concentration against the Sf9 cell lines stably expressing either receptor variant Ra or Rb. Wild type Sf9 cells were also tested as a negative control. The mean response from four wells receiving the same treatment in the same plate was regarded as one replicate, and three to four replicates from independent assays on different cell plates were analyzed (mean + SEM). Peptide and species abbreviations: MM, myomodulin; NmU, neuromedinU; PK, pyrokinin; ETH, ecdysis triggering hormone; PBAN, pheromone biosynthesis activating neuropeptide; DH, diapause hormone; Dm, *Drosophila melanogaster*; Hz, *Helicoverpa zea*; Hh, *Halyomorpha halys.*

NmU/PRXamide ligands from slug (ten peptides), insect (eight peptides), and mammals (two peptides) were tested against the two slug receptors stably expressed in Sf9 cells. To determine the binding strength of the peptides to the receptors, fluorescence intensity induced by the calcium influx into the cell was measured (Fig. 2). In the initial tests using 500 nM concentration of each ligand, two slug peptides, QPPLPRYa and QPPVPRYa, evoked distinct binding to both variants, as did AVPRPRIa. However, the other PRXamide peptides from the slug failed to trigger a significant response to either receptor (Fig. 2). The insect PRX peptides and the mammalian NmU and FMRF peptides tested in this study failed to show any significant binding activities compared to the control (Fig. 2). Between the two receptor variants, variant Rb was always more responsive to the peptide ligands than variant A. The empty Sf9 control cells had no activity (Fig. 2).

#### Responses of the natural peptides

**Figure 3:**
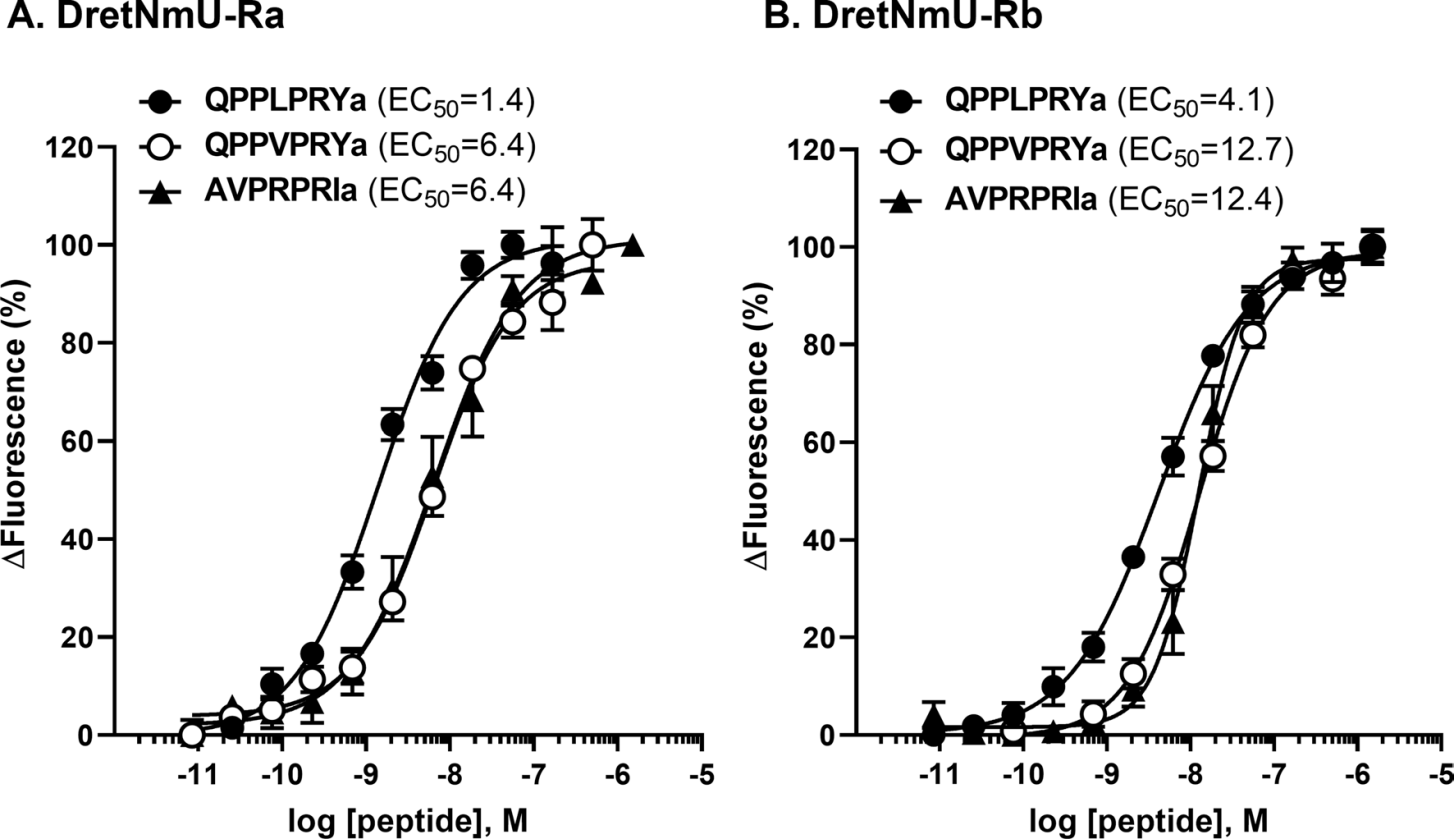
Normalized concentration-responses and EC_50_ values (nM) of the two NmU receptor variants, DretNmU-Ra (**A**) and DretNmU-Rb (**B**), from *Deroceras reticulatum* to the three bioactive peptides that elicited high responses in the screening experiment. Data represent the mean  ± SEM response of cells from four or five independent experiments. The mean response from four wells receiving the same treatment in the same plate was regarded as one replicate, and four to five replicates from independent assays on different cell plates were analyzed (mean ± SEM).

The three most active peptides (QPPLPRYa, QPPVPRYa, and AVPRPRIa) were serially diluted and evaluated for dosage dependent binding to DretNmU-Ra and DretNmU-Rb (Fig. 3). They are all heptapeptides and share the -PRXamide. DretNmU-Rb was more active to the peptide ligands than DretNmU-Ra. The maximum binding activity of receptor variant Rb was twice that of receptor variant Ra for the peptide ligands tested, although the EC_50_ values of the receptor variant Ra is lower than those of the variant Rb.

#### Responses of the modified peptides

**Figure 4:**
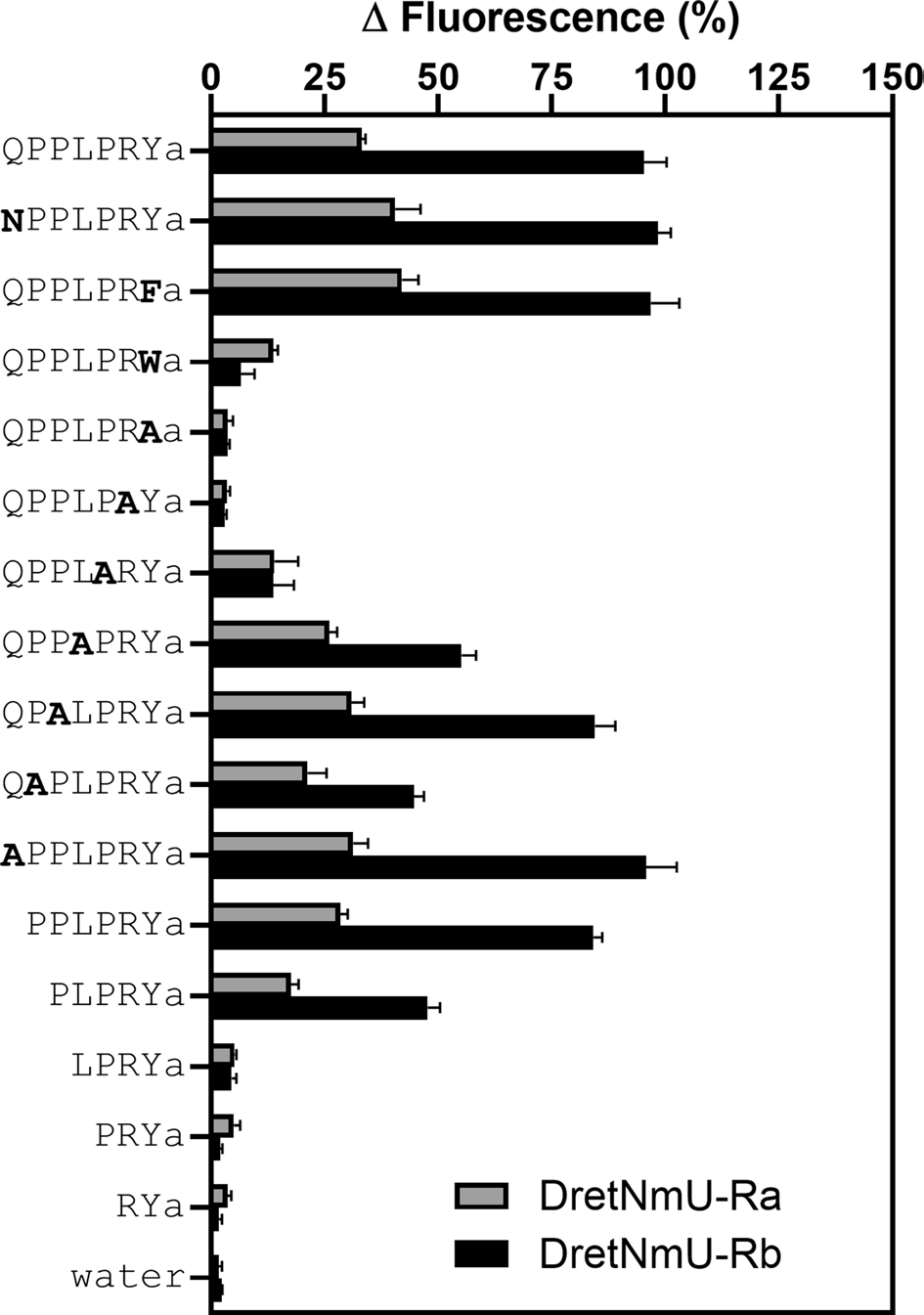
Structure–activity test for two receptor variants identified from *Deroceras reticulatum*. Synthetic peptides modified at various positions were assayed at 500 nM concentration against the Sf9 cell lines stably expressing either the receptor variant Ra or Rb. Amino acid substitutions compared to the true peptide ligand sequence (QPPLPRYa) are highlighted in bold. N, asparagine; F, phenylalanine; W, tryptophan; A, alanine. The mean response from four wells receiving the same treatment in the same plate was regarded as one replicate, and three to four replicates from independent assays on different cell plates were analyzed (mean + SEM).

To determine the core amino acids in the peptide ligand, the most active peptide (QPPLPRYa) was modified by replacing different amino acids or by removing one amino acid from the N-terminus. The various peptides were synthesized and tested at 500 nM concentration for binding to DretNmU-Ra and -Rb (Fig. 4). The glutamine (Q) at the N-terminus of the peptide was replaced with asparagine (N), which has the same polar and uncharged R group, NPPLPRYa. The binding strength to both receptors was similar to the natural ligand. The tyrosine (Y) at the C-terminus of the peptide was replaced with phenylalanine (F) or tryptophan (W), which have a similar aromatic amino acid group, QPPLPRFa or QPPLPRWa. The QPPLPRFa peptide showed a similar binding response to the receptors as the original ligand, but the QPPLPRWa peptide was dramatically reduced in its binding activity (Fig. 4). Each of seven amino acids (Q_1_P_2_P_3_L_4_P_5_R_6_Y_7_) of the natural ligand was replaced in series with alanine (A). When the P_5_, R_6_, or Y_7_ were replaced with alanine (A), these modified peptides almost lost their ability to bind the receptors. Interestingly, when the other amino acids, Q_1_, P_2_, P_3_ or L_4_, were replaced with an A in the natural ligand, they were still able to bind the receptors. Finally, with serial deletions of amino acids from the N-terminus of the natural ligand a gradual decrease of the binding ability was observed until the pentapeptide, PLPRYa, but then all activity was lost going from the tetra-peptide (LPRYa) to the di-peptide (RYa) (Fig. 4).

### Gene expression analysis

#### NmU/PRXamide peptide-encoding gene expression

**Figure 5:**
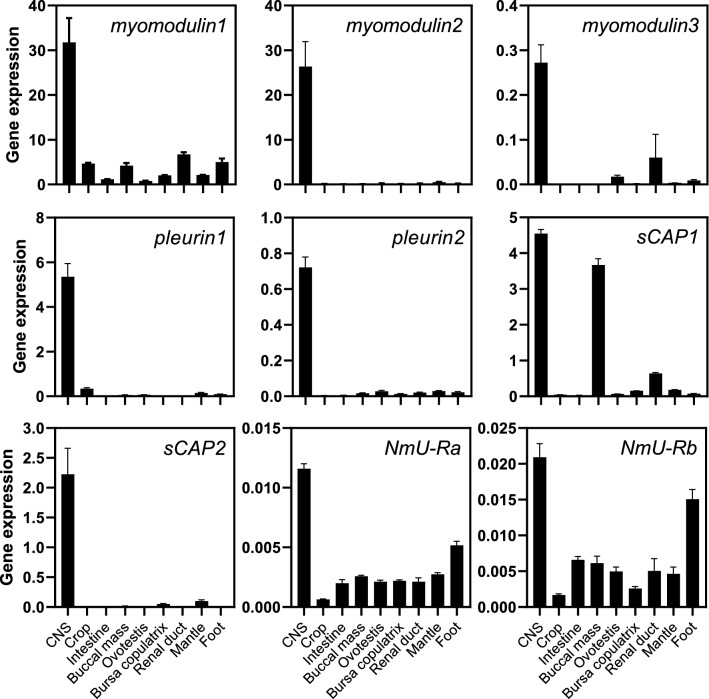
Expression analysis of the NmU/PRXamide peptide-encoding genes and the two receptor variants-encoding genes in different tissues of *Deroceras reticulatum*. Nine different tissues were dissected from adult slugs. Total RNAs were extracted from the tissues, RNA concentration was adjusted to 100 ng/μl, and cDNAs were synthesized from 1 μg of total RNAs. Quantitative real-time PCR (qRT-PCR) was conducted with cDNA templates from the different tissues using the SYBR Green method, and specificity was confirmed by melt curve analysis. Primer efficiencies were calculated from a cDNA template dilution series. The 26S proteasome regulatory subunit 8 (*rpt6*) was used as a reference gene. Three biological replicates per tissue and two technical replicates per reaction were analyzed. CNS, central nervous system.

The peptide-encoding genes, including *myomodulins*, *pleurins* and *sCAPs*, tested in this study were highly expressed exclusively in the CNS, compared to the other tissues in the slug (Fig. 5). The *myomodulin1* and *myomodulin2* genes were expressed at relatively higher levels than the other peptide-encoding genes and at about 100-fold greater than *myomodulin3*. The expression level of *pleurin1* was almost eightfold greater than *pleurin2*. The expression level of *sCAP1* was twice that of *sCAP2* in the CNS. Interestingly, the *sCAP1* gene also had high expression levels in the buccal mass of the slug.

#### Receptor gene expression

The receptor genes were also highly expressed in the CNS, followed by expression levels in the foot, whereas the expression levels were relatively lower in other tissues (Fig. 5). The receptor DretNmU-Rb was expressed at levels two to threefold greater than the -Ra in all tissues tested in general. Interestingly, the DretNmU-Rb receptor was highly expressed in the foot compared to the other tissues except for the CNS.

### Bioassay of the peptides in the slug

**Figure 6:**
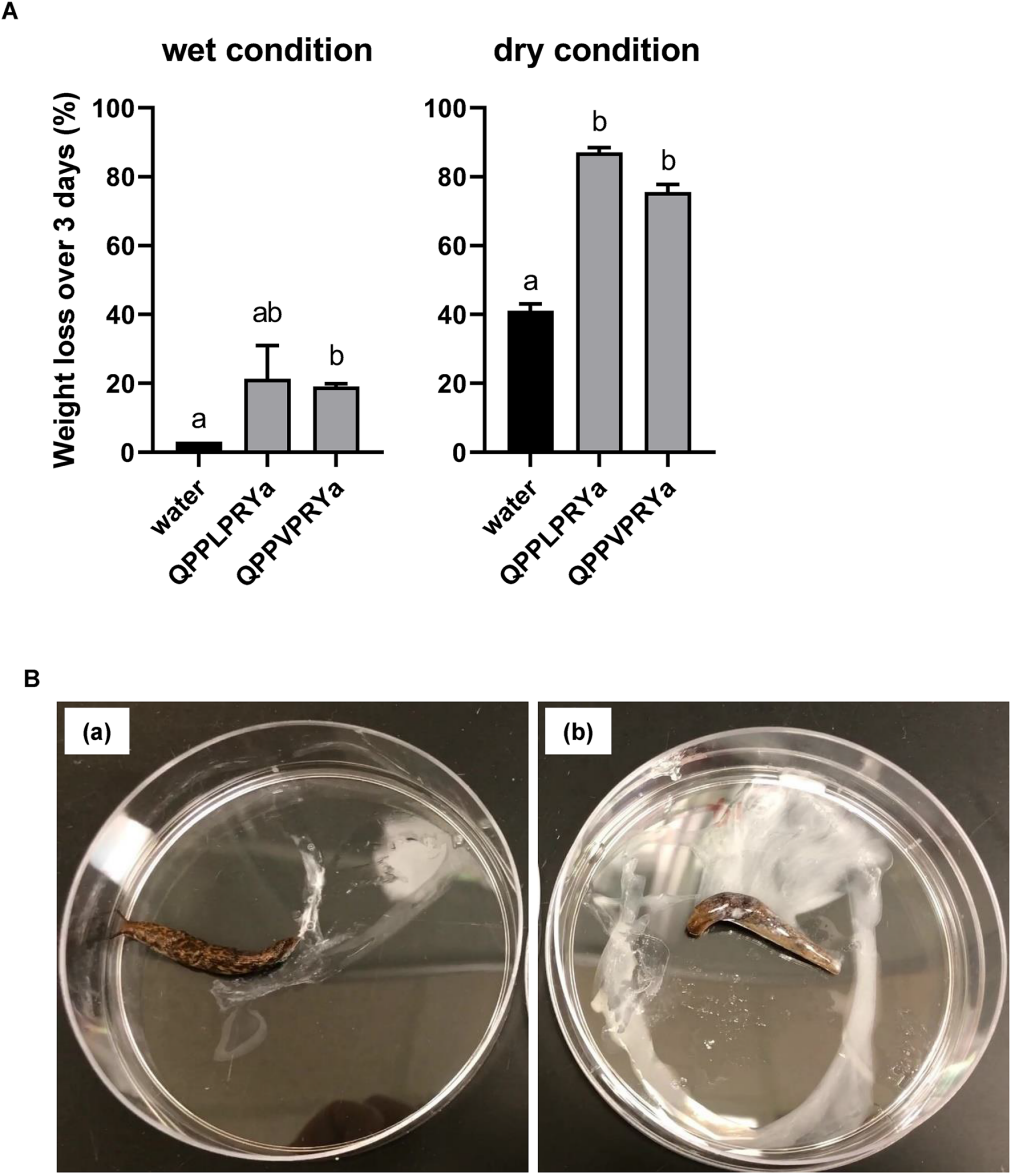
Injection bioassays of the NmU/PRXamide peptides for *Deroceras reticulatum*. (**A**) Effects of two NmU/PRXamide peptides on the weight loss (mean + SEM) of the slug (n = 4). Injection of 10 nmol of the peptide dissoved in 5 μl water using a Hamilton syringe and resulted in a loss of weight in both wet and dry conditions, although the effect was more clearly demonstrated under dry conditions. Different letters denote significant differences (P < 0.05) between treatments and control determined by One-way ANOVA followed by Dunnett’s multiple comparisons test using GraphPad Prism version 8.0. (**B**) Injection of the peptide (either QPPLPRYa or QPPVPRYa) triggered a series of distinct, atypical behaviors such as initial rapid crawling, flaring of the mantle, repeated retraction and extension of the ocular tentacles, hyperextension of the ocular tentacles, abnormal body shapes, and secretion of copious milky mucus (see the video in Supplementary_Data_2.mp4). (a) water injection and (b) peptide injection.

Based on the functional tests and response to the receptors above, we selected the two most active peptide ligands, QPPLPRYa and QPPVPRYa, and investigated their in vivo effects on *D. reticulatum*. When the peptides were injected, the test slugs compared to the control slugs showed a series of atypical behaviors within 30 min: the slugs displayed very rapid crawling, flaring of the mantle, rapid retraction and extension of the ocular tentacles, hyperextension of the ocular tentacles, abnormal body shapes, and immobilization. In addition, after 3 days some slugs died (QPPLPRYa: dry conditions—100% mortality; wet conditions—50%; QPPVPRYa: dry conditions—50%; wet conditions—0%). Furthermore, the slugs injected with the peptides secreted large amounts of milky mucus, causing weight loss. None of the control slugs displayed these behaviors, other than secreting a small volume of milky mucus after injection. The treated slugs that were kept in a wet environment ultimately showed mild weight loss, while those kept under dry conditions, showed substantial weight loss (Fig. 6 and Supplementary_Data_2.mp4). The relative weight losses of the injected slugs over 3 days were greater than twice that of the control slugs treated with water and kept under wet or dry conditions.

## Discussion

Neuromedin U (NmU) is a bioactive peptide originally isolated from the porcine spinal cord^1^ and is highly conserved among vertebrates, including humans^3^. Since the *Drosophila* genome has been released^27^, insect PRXamide family peptides and their receptors have been characterized as vertebrate NmU orthologs and NmU receptors (NmU-Rs) due to their sequence similarity^4,9–11,28–30^. In evolutionary lower animal groups such as nematodes, the NmU/PRXamide orthologs and receptors have also been identified from *Caenorhabditis elegans*^20^.

The first molluscan NmU and PRXamide orthologs have been reported in a terrestrial snail^21^. The gene structure and expression of the NmU and PRXamide orthologs in the terrestrial snail are similar to the slug *pleurin1* and *pleurin2* genes expressed in the central nervous system in our studies^26^ (Figs. 5 and S3). These peptides have also been considered as homologs of the peptides produced by the *hugin*, *capa*, and *eth* genes in *Drosophila*^31^. However, little is known concerning the molluscan receptors for the NmU and PRXamide family peptides. In this study, we utilized the slug transcriptome to identify a complete list of genes encoding NmU and PRXamide peptides and determined their ability to activate NmU-Rs identified in *D. reticulatum*. Then, we deorphanized the receptors with three natural ligands, QPPLPRYa, QPPVPRYa, and AVPRPRIa, encoded from two *myomodulin* genes. This is the first report on the molluscan NmU-R and its agonistic peptide ligands.

The slug NmU-Rs contain seven transmembrane (TM) domains, indicating that the receptors belong to the typical G protein-coupled receptor (GPCR) family. The conserved ERY motif found next to TM3 is modified to DRY in this receptor (Fig. S2). Two transcript variants (DretNmU-Ra and DretNmU-Rb) of the receptor gene are derived by alternative splicing events that occur at the interface between exon 4 and exon 5. Due to a frame shift within the 11-bp intron region in variant Rb, the two variants contain a different intracellular segment (about 40 aa) at their C-termini. As shown in the protein sequences, the two GPCRs are mostly conserved, except for their C-terminal intracellular end. The remaining protein sequence, including the seven TM domains and extracellular domains, are derived from exons 1 through 4, indicating the 3-dimensional structures and binding sites should be similar between two GPCR variants. The extracellular domains of GPCRs are critical for maintaining the binding pockets to receive their specific ligands. The GPCR structures of the insect PRXamide peptides have been characterized through molecular site-directed mutations, 3D-modeling, and exchanging extracellular domains for the binding interactions and signaling^32–37^.

The variable sequences of the intracellular C-terminal regions should not affect the GPCR 3D-structure. Therefore, the C-terminal intracellular part (40 aa) presumably contributes to the discrepancy between the ligand-receptor binding strength of the two GPCR variants. The intracellular domain at the C-terminal end is expected to activate G proteins, opening specific calcium channels necessary for the influx of Ca^2+^ as a secondary messenger, triggering a signal transduction cascade^10,15^. The responses of DretNmU-Rb to the ligands tested in this study were always more active (~ twofold) than those of the variant Ra. This suggests that the C-terminus intracellular domain may contribute to the conformational transition of the GPCR to activate G proteins and increase the Ca^2+^ influx, once the ligand binds to the receptor. In insect PBAN GPCRs, the C-terminus intracellular sequences are functionally significant for ligand-induced receptor internalization to regulate the strength and duration of receptor-mediated cell signaling^38–41^. Further studies on the intracellular components linked to GPCR signal transduction are needed to better understand these processes in slugs.

In arthropods, multiple PRXamide peptides are often encoded from the same gene, but generally members from different families of peptides are not encoded within a gene^4,42,43^. However, the slug *myomodulin2* gene produces peptides from two different peptide families, Myomodulin (MM) (-MLRLa) and NmU/PRXamide (Table 1 and Fig. S1). This is similar to what has been observed in *Aplysia* where a single gene was found to encode not only the canonical MM peptides but also three NmU/PRXamide peptides (called MMG2-derived peptides; MMG2-DPs) including QPPLPRYa (MMG2-DPb), AVALPRIa (MMG2-DPd), and AVPRPRIa (MMG2-DPf)^44^. Interestingly, both QPPLPRYa and AVPRPRIa are considered natural ligands of the NmU receptor in *D. reticulatum* (Table 1, Fig. 3), suggesting these peptides are highly conserved at least in Gastropoda.

Although the *myomodulin3* gene encodes only NmU/PRXamide peptides, its DNA sequence is similar to the *myomodulin1* and *myomodulin2* genes, although the *myomodulin3* encodes all three NmU/PRXamide peptides in this study. The canonical MM peptides were originally isolated from a feeding muscle in *Aplysia*^45^ and the gene encoding the MM peptides was cloned later^46,47^. More *myomodulin* genes and peptides have been reported from molluscan species either from transcriptomes/genomes or from proteomics^26,48,49^.

*Pleurin* genes and *sCAP* genes produce three and two NmU/PRXamide peptides, respectively. Due to their high sequence similarity to insect PRXamide peptides, they have been characterized as orthologs of insect pyrokinin or CAPA peptides^6,31,50,51^. However, no functional studies have been reported yet. If additional receptors are identified from the slug, they could be deorphanized by these peptides.

The natural ligand’s structural modification stuides indicate that the C-terminal three amino acids (PRXamide) comprise a core motif for the ligand-receptor binding. When any of the three amino acids were replaced by the simplest non-polar and aliphatic amino acid alanine (A), as in QPPLPRAa, QPPLPAYa, and QPPLARYa, the receptor response to the ligands almost completely disappeared. When the C-terminal tyrosine (Y) was replaced by aromatic and non-polar phenylalanine (F) or tryptophan (W), QPPLPRFa retained normal activity to the receptors, but QPPLPRWa activity was eliminated. Interestingly, all three amino acids, Y, F, and W, have the aromatic functional group, and tryptophan (W) only contains an indole side chain that could interfere with the ligand–receptor binding. Additional structural–activity relationships developed through ligand modification or mimics will be helpful for finding the core residues in the ligands and developing potential targets for pest control^52,53^.

In contrast, the N-terminal glutamine (Q) in the peptide did not have an effect on ligand–receptor binding. For example, when Q was replaced with asparagine (N) or alanine (A), or just eliminated from the ligand, there was no significant decrease in the ligand-receptor binding activity. However, the second and fourth amino acids from the N-terminus seem to be more critical, because when they were replaced with alanine (A), binding activity decreased about 42–53% for the variant Rb and about 22–36% for the variant Ra, whereas the third position proline (P) between these two essential amino acids was not critical for binding activity.

A minimum peptide length appeared to be critical for ligand–receptor binding; at least five amino acids with the core motif (PRXamide) were found to be essential to activate the receptors. From the overall structural analyses, it seems that the core C-terminal three amino acids (PRXamide) and a minimum five amino acids are critical to activate the slug receptors and trigger signal transduction. It is also consistent in different animal groups in general^4^. More studies on peptide 3D modelling and additional structural relationships would help to elucidate the agonistic and/or antagonistic effect on the target receptor. It is noteworthy that the families of insect PRXamide peptides and the mammalian NmU peptide tested were not able to activate the slug receptors (Fig. 3). It would be very interesting to examine the activity of slug peptides with insect PRX-Rs or vertebrate NmU-Rs.

In vertebrates, the NmU peptides function in the activation of ion transport and contractile activity in intestinal and arterial musculature via NmU-R^3^. In insects, many PRXamide peptides have various functions, including heartbeat modulation, visceral muscle contraction, (anti)diuresis, pheromone biosynthesis, cuticle melanization, etc^54–58^. In *Aplysia*, immunocytochemistry studies on two MM peptides (original names, MMs and myomodulin gene2-derived peptides) indicate that the two *myomodulin* genes are differentially expressed in the buccal ganglion, the buccal musculature, and in the pedal ganglion. These organs are involved in multiple actions including feeding and muscle contractions, suggesting that these peptides play a broad role in feeding behavioral plasticity^44^. The gene expression studies of various organs in *D. reticulatum* showed that the active peptide-encoding genes (*myomodulins* and *pleurins*) are highly expressed in the CNS, whereas the receptor gene (DretNmU-Rb) was highly expressed in both CNS and foot. The results are similar to the studies of *Aplysia*.

In the injection bioassays, the NmU/PRXamide peptide triggered atypical behaviors in the slug, including rapid movement, abnormal body shapes, flaring of the mantle, and rapid retraction and extension of the ocular tentacles, which might be derived from excessive muscle contractions. This likely immoderate muscle contraction in the slug induced by the peptide is a similar physiological change observed in the insects above. It is noteworthy that treated specimens secreted copious amounts of milky mucus, which is a well-known defense response for *D. reticulatum* usually against physical attack^59–62^. This suggests that the pedal mucus gland could be an additional location for receptors, which active peptides could directly or indirectly target. It will be interesting to perform more detailed investigations to gain a better understanding of how slugs respond to the peptides and the underlying mechanisms involved, which will increase our knowledge of the physiological function of the peptides and aid in the development of novel pesticides against molluscan pests.

## Methods

### Slug

*Deroceras reticulatum* species were collected in the Willamette Valley, Oregon, USA, and maintained in a humid container with carrot and lettuce in a controlled incubator (18 °C, 90% RH, 12:12 = light:dark, dim light).

### Nucleic acids isolation

Mucus was removed by scraping the surface of live slugs using sterilized forceps before homogenization. A mature slug was homogenized in ATL buffer provided in the QIAamp DNA Mini Kit (Qiagen, USA) using a PYREX glass pestle grinder (Corning, USA). The slimy homogenate was diluted with additional ATL buffer, further homogenized and the homogenate was pooled in a microcentrifuge tube. The genomic DNA was isolated using QIAamp DNA Mini Kit according to the manufacturer’s instructions, and stored at − 20 °C until use. Three mature slugs with mucus-removed were individually homogenized in 1 ml lysis buffer provided in the PureLink RNA Mini Kit (Invitrogen, USA) using a PYREX glass pestle as above. The slimy homogenate from the three samples was diluted with additional lysis buffer, further homogenized and pooled into a 15 ml-Falcon tube. Total RNA was extracted from the combined sample using the PureLink RNA Mini Kit following the manufacturer’s instructions, quantified on a NanoDrop2000 spectrophotometer (Thermo Fisher Scientific), and stored at − 80 °C.

### cDNA synthesis

For the random amplification of the cDNA ends (RACE-PCR), 5′- and 3′-RACE-ready cDNAs were synthesized from 1 µg of total RNA (see above) using the SMARTer RACE 5′/3′ Kit (Clontech, USA) according to the manufacturer’s instructions. For conventional PCR, first-strand cDNAs were also synthesized from 1 µg of total RNA using the SuperScript IV First-Strand Synthesis System (Invitrogen) according to the manufacturer’s instructions. Both cDNAs were stored at − 20 °C until use.

### BLAST search

We have previously reported seven PRXamide-encoding genes including *myomodulin1*, *myomodulin2*, *myomodulin3* (partial), *pleurin1*, *pleurin2*, *sCAP1*, and *sCAP2* in the *D. reticulatum* transcriptome^26^. For the NmU/PRXamide receptor genes, BLAST searches against the slug transcriptome found a partial transcript (c90580) that was most similar to the mammalian NmU-Rs and insect PK/CAPA receptors. The full-length sequences of these genes were amplified using a combination of RACE-PCR and conventional PCR, cloned, and sequenced. (see Supplementary Data 1 for detailed methods).

### Expression vector construction

The full-length sequence of the receptor gene was PCR amplified from the previously cloned-pJET1.1 vector constructs using Phusion DNA Polymerase (Thermo Fisher Scientific) with a sense primer, including the Kozak sequence (GCCACCATGG; underlined ATG = start codon), and an anti-sense primer including the stop codon. PCR was performed under the following conditions: 98 °C for 30 s; 35 cycles of 98 °C for 10 s, 55 °C for 20 s, and 72 °C for 1 min; then 72 °C for 10 min. Primer sequences used in this study are listed in Table S1. PCR products were purified using the GeneJET Gel Extraction Kit (Thermo Fisher Scientific), and ligated into the pIB/V5-His TOPO-TA expression vector (Invitrogen). The vectors containing the receptor genes were sequenced and the orientation of the sequences was confirmed.

### Expressing receptors in Sf9 cells

Sf9 cells were cultured in a 50 ml-Erlenmeyer flask while shaking at 145 rpm at 28 °C using Insectagro Sf9 Medium (Corning) supplemented with 5% heat-inactivated fetal bovine serum (HyClone, USA) and antibiotics (penicillin and streptomycin; Gibco). Three µg of either pIB/DretNmU-Ra or pIB/DretNmU-Rb plasmid was added to 100 μl of serum-free medium, and 15% CellFectin II (Invitrogen) in 100 μl serum-free medium was prepared, and both were placed at room temperature for 5 min. Samples were combined by pipetting and placed at room temperature for another 20 min. The plasmid-CellFectin II mixture was then added to a T-25 flask containing a layer of Sf9 cells covering about 70% of the surface, and incubated overnight. The next morning, the medium was replaced with fresh medium containing 50 μg/ml blasticidin. The medium was replaced daily with 50 μg/ml blasticidin containing medium for 1 week, and then the blasticidin concentration was gradually reduced to 30 μg/ml for the next 2 weeks. When culture was confluent, the recombinant cells were lifted and transferred to suspension cultures and grown until the exponential growth phase, and then stock cultures were frozen in liquid nitrogen. These frozen stock cell lines were thawed, cultivated for several passages in media containing 20 μg/ml blasticidin, and used for the ligand binding assays.

### Peptide ligands

Analysis of the *D. reticulatum* transcriptome for neuropeptides^26^ revealed seven genes encoding putative PRXamide peptides (Table 1). For confirmation, the full-length sequences were PCR amplified, cloned, and Sanger sequenced (details above). In particular, the partial transcript of *myomodulin2* gene was extended by RACE-PCR to identify 5′- and 3′-untranslated regions (see details above). With experimentally confirmed full-length sequences, the putative peptide sequences were predicted according to the mono- and dibasic cleavage site rules^51^. Among twenty-six different peptides amidated in their C-terminal ends, eight representative slug peptides were selected and synthesized by Peptide 2.0 (Chantily, USA). Several insect PRXamide peptides and vertebrate peptides (FMRF amide and Neuromedin U) were also synthesized for comparison.

### Functional testing of receptors

Two days before the binding assay, ~ 50,000 Sf9 cells expressing the DretNmU-Ra or -Rb from suspension cultures were dispensed into each well of a black 96-well plate (Corning C3603) and incubated at 28 °C overnight. At 48 h, cells were rinsed once with 100 μl of fresh medium without FBS. After removing the media, cells were incubated with 95 μl of 1 × FLIPR Calcium 6 reagent (Molecular Devices, USA) containing 2.5 mM probenecid at room temperature in the dark for 1 h. The Calcium 6 reagent-loaded cells were transferred to the Flexstation 3 multi-mode microplate reader (Molecular Devices) to measure fluorescence change (∆fluorescence) before and after loading 5 μl of peptide ligands (20×), detecting emission at 535 nm with excitation at 485 nm. Fluorescence measurements from each well on the column were taken every 5 s for 4 min. The peptide ligand was added by automatic pipettor once at 30 s from the beginning, and 5 μl of 5 μM ionomycin was added at 3 min to confirm calcium activity. Baseline fluorescence was determined by averaging 5 time points from each well prior to treatment with ligand and the resulting response was expressed as a percent increase in fluorescence relative to the baseline value.

For the initial screening and structure–activity tests, each cell line was exposed to a single concentration of 500 nM of each peptide or control. For the dosage-response assay, threefold serial dilutions of selected ligands with water were used, giving final ligand concentrations between 8 pM and 1.5 μM. The mean response from four wells receiving the same treatment in the same plate was regarded as one replicate, and at least three replicates from independent assays on different cell plates were analyzed. Data were transformed into a log scale and fit into the non-linear regression curve. EC_50_ values were obtained by statistical analysis using the sum of squares F-test function using GraphPad Prism version 8.0 (San Diego, CA, USA).

### Quantitative real-time PCR

Quantitative real-time PCR (qRT-PCR) was conducted using the SYBR Green method in a StepOnePlus Real-Time PCR System (Applied Biosystems). Nine different slug tissues, such as central nervous system (CNS), crop (without content), intestine (with digestive gland), buccal mass, ovotestis, bursa copulatrix (with albumen gland), renal duct, mantle and foot, were dissected, homogenized and extracted for total RNAs (see above) with three biological replicates. cDNA templates were synthesized from 1 μg of total RNA using Verso cDNA Synthesis Kit with oligo dT/random hexamer primers (ThermoFisher Scientific) according to the manufacturer’s instructions. The qRT-PCR reaction conditions were performed at 95 °C for 10 min; 40 cycles of 95 °C for 15 s and 60 °C for 1 min; followed by a melting curve analysis over the range of 60–95 °C with 0.3 °C/min increments, with the specific primers (Table S1). Six different concentrations of cDNA pool were used to construct a standard curve for each primer set to determine primer efficiency. Six candidate reference genes (*β-cop, rpl3, rpl40, rpt6, vha26*, and *vps16*) were evaluated for their primer efficiency and variability through pilot tests, then the 26S proteasome regulatory subunit 8 (*rpt6*) gene was selected as a reference gene (Table S2) (see Supplementary Data 1 for detailed methods).

### Bioassay of the peptides in the slug

Two peptides, QPPLPRYa and QPPVPRYa, were dissolved in purified water to obtain a 2 mM concentration and individually injected into the adult slugs (270–520 mg in weight). A Hamilton syringe was used to inject a dose of 10 nmol peptide in 5 μl water or water only for control into the posterior part of the foot of the slug (n = 4). The foot was chosen as the injection site in order to avoid possible damage to internal organs, which are primarily located underneath the mantle. The injected slug was observed for an initial 30 min and notes were taken on its atypical behavior compared to the control, e.g. rapid movement, abnormal body shapes, flaring of the mantle, rapid retraction and extension of the ocular tentacles, and excessive milky mucus secretion. Since the treated slugs secreted a large amount of the defensive mucus, weight loss was assessed by weighing the slugs on a VWR B2-Series Analytical Balance immediately before the treatment and again 3 days later. Post treatment weights were obtained after slugs were placed under wet conditions (slugs placed in a ventilated 8 oz plastic container lined with damp tissue paper) or under dry conditions (same container type without damp tissue paper). Slugs were placed under these conditions approximately 30 min after injection. These plastic containers were maintained in a growth chamber (Thermo Scientific Precision Model 818) at 18 °C and 12 h photoperiod. Statistical analysis was performed by One-way ANOVA followed by Dunnett’s multiple comparisons test using GraphPad Prism version 8.0 (GraphPad Software, CA, USA).

## Supplementary Information

Supplementary Information 1.

Supplementary Information 2.
