# Supplementary material for: Identification and functional characterization of the first molluscan neuromedin U receptor in the slug, Deroceras reticulatum
Source: Sci Rep. 2020 Dec 18;10:22308. doi: 10.1038/s41598-020-79047-x (PMC7749107; doi:10.1038/s41598-020-79047-x)
Supplement: Supplementary file 1 — Supplementary Information 1. [file 41598_2020_79047_MOESM1_ESM.pdf]

Supplementary data 1

Identification and functional characterization of the first molluscan neuromedin U receptor in the slug, *Deroceras reticulatum*

Seung-Joon Ahn[1,2], Rory Mc Donnell[3], Jacob A. Corcoran[1,4], Ruth C. Martin[5], Man-Yeon Choi[1*]

[1]USDA-ARS, Horticultural Crops Research Unit, Corvallis, Oregon, USA; [2]Department of Biochemistry, Molecular Biology, Entomology & Plant Pathology, Mississippi State University, Mississippi State, Mississippi, USA; [3]Department of Crop and Soil Science, Oregon State University, Corvallis, Oregon, USA; [4]USDA-ARS, Biological Control of Insects Research Unit, Columbia, Missouri, USA; [5]USDA-ARS, Forage Seed and Cereal Research Unit, Corvallis, Oregon, USA

*Email to Man-Yeon Choi (man-yeon.choi@usda.gov)

Methods

Rapid amplification of cDNA end (RACE), PCR and molecular cloning of full-length cDNAs

The RACE strategy was used to amplify both 5'- and 3'-ends of the partial transcript sequence found in the slug transcriptome. With a gene-specific primer binding the partial sequence and a universal primer binding to the adaptor sequence added to the RACE-ready cDNAs, both ends of the partial sequence were amplified using Phusion High Fidelity DNA polymerase (Thermo Fisher Scientific) under the following conditions: 98 °C for 30 s; 35 cycles of 98 °C for 10 s, 68 °C for 20 s, and 72 °C for 1 min; then 72 °C for 10 min using a Veriti 96-Well Fast Thermal Cycler (Applied Biosystems, USA). Based on the identified sequences at both ends, the open reading frame was defined. The full-length coding sequence was amplified from the first-strand cDNA using Phusion High Fidelity DNA polymerase under the following conditions: 98 °C for 30 s; 35 cycles of 98 °C for 10 s, 55 °C for 20 s, and 72 °C for 1 min; then 72 °C for 10 min. Primer sequences used in this study are listed in Table S1. PCR products were run in a 1.2% agarose gel, purified using the GeneJET Gel Extraction Kit (Thermo Fisher Scientific), and cloned into a pJET1.2 vector using the CloneJet Cloning Kit (Thermo Fisher Scientific) for sequencing. The sequencing results were analyzed using Geneious 8.1 software (Biomatters, USA).

Quantitative real-time PCR (qRT-PCR)

cDNAs were synthesized from 1 μg of total RNA using Verso cDNA Synthesis Kit with oligo dT/random hexamer primers (ThermoFisher Scientific) according to the manufacturer's instructions. The same cDNA preparation for each RNA sample was performed without reverse transcriptase as negative controls. Synthesized cDNAs were stored at -20 °C until use. Quantitative real-time PCR (qRT-PCR) was conducted with different cDNA templates using the SYBR Green method in a StepOnePlus Real-Time PCR System (Applied Biosystems). Primers for target and reference genes are listed in Table S1. The qRT-PCR reaction mixture was prepared in optical 96-well plates with a 20 μl-volume containing 10 μl FAST SYBR Green Master Mix, 1 μl cDNA template, 1 μl primer pair (5 μM each), and 8 μl nuclease-free water. Reaction conditions were: 95 °C for 10 min; 40 cycles of 95 °C for 15 s and 60 °C for 1 min; followed by a melting curve analysis over the range of 60 - 95 °C with 0.3 °C/min increments. Six different concentrations of cDNA pool were used to construct a standard curve for each primer set to determine primer efficiency. Three biological replicates were performed using different preparations of cDNA and the average of the replicate values was calculated to determine the final efficiency. Six candidate reference genes (*β-cop, rpl3, rpl40, rpt6, vha26*, and *vps16*) were evaluated for their primer efficiency and variability through pilot tests, then the 26S proteasome regulatory subunit 8 (*rpt6*) gene was selected as a reference gene (Table S2). Due to the high sequence similarity, the variant-specific primers were designed using the variable sequences (Supplementary Data).

Table S1. Primers used in this study.

Target gene	Use	Primer name	Sequence (5' -> 3')	Length (bp)
myomodulin1	3'-RACE	DretMmd1-rF1	AAGCTAGCCCTACCAGAACAGAAGCCGT	28
	5'-RACE	DretMmd1-rR1	TCGTTGAGCAACAAGAGACGGGGCAAC	27
	full-length	DretMmd1-F1	AAGAACCATGCATCGTCGAG	20
	full-length	DretMmd1-R1	GCTTATCTACAGAGCTGATCTC	22
	qRT-PCR	DretMmd1-qF1	TTTCCGACATAGACGCTCGG	20
	qRT-PCR	DretMmd1-qR1	TTCAGGTCAACGGGTTCCAG	20
myomodulin2	3'-RACE	DretMmd2-rF1	CGCAGCTAGGAACAGCATTTTAGGAAGCA	29
	5'-RACE	DretMmd2-rR1	GCGGTCAAGGCTCTCCACGGGC	22
	full-length	DretMmd2-F1	TACATTCATAGCAGGCCTACAC	22
	full-length	DretMmd2-R1	TTGTTTATTTAGACCAACTTGGC	23
	qRT-PCR	DretMmd2-qF1	ATTTGACCAGCCCAGGGATG	20
	qRT-PCR	DretMmd2-qR1	CAGTCATGGCGTCATCCAGA	20
myomodulin3	3'-RACE, full-length	DretMmd3-rF1	ACGCTAGTGCAATGGGAATCTCAGAAGCG	29
	5'-RACE	DretMmd3-rR1	GCCATCCGATGACCGTCCCTGGC	23
	full-length	DretMmd3-R1	CATTTACGCCCAATCATATGGC	22
	qRT-PCR	DretMmd3-qF1	GCGCATCTCGACCATGAAAC	20
	qRT-PCR	DretMmd3-qR1	AAGTAGCAGCCACTGAAGCC	20
pleurin1	3'-RACE	DretPleurin1-rF1	CCGCGAATTGGACGACGGGATGCCAC	26
	5'-RACE	DretPleurin1-rR1	TCCCCACTCGATGCCTCCTCACTGCCT	27
	full-length	DretPleurin1-F1	AAGGCAACAACAGACGGACAAATAACT	27
	full-length	DretPleurin1-R1	CGAAGCAGCTGCAGGAAAGGC	21
	qRT-PCR	DretPleurin1-qF1	TGGAAATAAGGAAGGAAATAGCGGA	25
	qRT-PCR	DretPleurin1-qR1	CGTCTTTGTCTACATCGAACTTCC	24
pleurin2	3'-RACE	DretPleurin2-rF1	ACCCGCGCATTGGACGACGGGAAAC	25
	5'-RACE	DretPleurin2-rR1	GCGACCTGTGCTGTGGGTAGCGACT	25
	full-length	DretPleurin2-F1	ATGCACCCTCCAATCAGCGT	20
	full-length	DretPleurin2-R1	AATGCCGTGTCAGTAGAGCG	20
	qRT-PCR	DretPleurin2-qF1	GCGTATTCACACAGGGTCCA	20
	qRT-PCR	DretPleurin2-qR1	CTCTCACTCGCGTAGTCGTC	20
sCAP1	3'-RACE	DretSCAP1-rF1	AACGCCGTGGGTGGGTCCGG	20
	5'-RACE	DretSCAP1-rR1	AGGATGGCTGGTGATGGGGTCCGT	24
	full-length	DretSCAP1-F1	AGAACAAGCTTAAACCAACCTC	22
	full-length	DretSCAP1-R1	GTCCGTCTATTTCCTGATTAGG	22
	qRT-PCR	DretSCAP1-qF1	CGTTCCCAGACAAAGTCCGA	20
	qRT-PCR	DretSCAP1-qR1	GGGCGGAGTTCCTCTTTACC	20
sCAP2	3'-RACE	DretSCAP2-rF1	TCGCTCCAGCCAGTCGCAACCAG	23
	5'-RACE	DretSCAP2-rR1	CCGTGGCAGCCGGGGTCATGG	21
	full-length	DretSCAP2-F1	TTGACCTGTCACAATGGAACTC	22
	full-length	DretSCAP2-R1	GCTGGGTGGGACGATGTTAC	20
	qRT-PCR	DretSCAP2-qF1	CAACGAGCGTCCTTCTCTGT	20
	qRT-PCR	DretSCAP2-qR1	AAGCCAAGTAGCCCGATCTG	20
NmU-R	5'-RACE	DretNmUR-rR1	TGGAAGCGCCAACAGTAGAGTCAAGACGT	29
	3'-RACE	DretNmUR-rF1	GGGCACCGTTCCATTCGCAGCG	22
	full-length	DretNmUR-F1	CAAGACTCTGACATGACTGACGGTAG	26
	full-length (Kozak)	DretNmUR-F2	GCCACCATGACTGACGGTAG	20
	full-length	DretNmUR-R1	GAGCTGATTTTCTTAGACAGTTGTGGAG	28
	qRT-PCR (-Ra)	DretNmUR-qF1	ACTACGAGTACAGCGAGGCA	20
	qRT-PCR (-Rb)	DretNmUR-qF2	AGTACAGGTGGGAAAGAGCG	20
	qRT-PCR	DretNmUR-qR1	TGTTTCTGGTCGTAACCCGG	20

Table S2. Primer efficiency test to select a reference gene for quantitative real-time PCR (qRT-PCR).

**Concentration (ng/µl)**	**20**	**10**	**2**	**1**	**0.2**	**0.1**	**Slope**	**Amplification factor**	**Efficiency (%)**
**Conversion (Log10)**	**1.3**	**1.0**	**0.3**	**0.0**	**-0.7**	**-1.0**			
βCop	24.9	26.1	28.3	29.7	31.4	32.6	-3.3	2.0	102.2
RpL3	21.9	22.8	25.2	26.3	28.6	29.9	-3.5	1.9	94.3
RpL40	21.2	22.3	24.7	25.8	27.9	29.6	-3.6	1.9	91.3
Rpt6	24.4	25.5	28.0	28.9	31.7	32.4	-3.5	1.9	91.9
Vha26	22.6	23.7	26.1	27.3	29.9	30.9	-3.6	1.9	89.1
Vps16	27.4	27.9	29.9	30.8	33.7	34.6	-3.2	2.0	104.3
myomodulin1	22.3	23.5	26.0	26.9	29.6	30.7	-3.6	1.9	88.8
myomodulin2	22.0	23.0	25.7	26.2	28.9	30.0	-3.4	2.0	95.4
myomodulin3	26.3	27.2	29.7	30.5	31.9	33.8	-3.1	2.1	109.7
pleurin1	23.9	24.9	27.4	28.3	30.7	31.8	-3.4	2.0	96.2
pleurin2	26.7	27.6	30.0	30.9	33.3	34.9	-3.5	1.9	93.8
sCAP1	23.6	24.7	27.3	28.3	31.0	31.7	-3.6	1.9	91.2
sCAP2	24.9	25.9	28.4	29.4	31.5	32.7	-3.4	2.0	98.8
NmU-R	28.1	27.8	30.8	30.7	33.6	37.0	-3.7	1.9	87.0

Table S3. Amino acid (AA) sequence identity of DretNmU-Ra and DretNmU-Rb from *Deroceras reticulatum* with PRXamide receptors from the fruit fly *Drosophila melanogaster*.

	**AA identity**	**1**	**2**	**3**	**4**	**5**	**6**
**1**	DretNmU-Ra	100					
**2**	DretNmU-Rb	88.3	100				
**3**	DmPK1-R	40.6	40.6	100			
**4**	DmPK2-R1	38.3	37.7	49.4	100		
**5**	DmPK2-R2	38.9	38.0	51.5	76.3	100	
**6**	DmCapa-R	36.8	37.1	38.0	33.6	32.5	100
**7**	DmETH-R	26.9	25.7	26.9	27.2	25.1	25.7

Myomodulin1 (395 aa)

*MHRRGLISLAVAVCLQLSLGYA*DNDASKANSDSSQDASLSRAKR**GGYDMLRL**GR**GLNMLRL**GKRMYDST
SDLNENTPSNWADLQDYVEHDPENVGFDYSTFPESLEDSIIQAQEGKYGKDLVDMGKR**KMSMLRL**GKRS
VDNFDEDASAHFRHRRSASSVGGAVASEVLQSTNQASKDLEPVDLKDEDDDELLVEYPNDISQSEEDIDG
KEWLPVISMGKR**QLSMLRL**GK**RSLGMLRL**GKRESEDDEEKR**ALGMLRL**GKR**QLSMLRL**GKR**SLGML**
RLGKRPSDDLDEVYGDEDDLTSEDGKR**AMSMLRL**GKR**PMSMLRL**GKR**PMSMLRL**GKR**PMSMLRL**G
KR**PMSMLRL**GKR**PMSMLRL**GKR**PMSMLRL**GKR**PMSMLRL**GKRDDEEKRDMSKRSAL

Myomodulin2 (218 aa)

*MNSLYETCIYFCAIAFIAGTSLVQA*KVESSRAETSANK**GQFSAARL**GR**GLQMLRL**GKRSVNLDSLNPDQAN
SHLTSNDVQAVLASIFDQPRDESRR**QPPLPRY**GRDSNNNVKGRLLDDAMTDNSGVYQADFFPLSSQR**FFF**
RPAPRGGRYRKSVPAGRLAYGSYISQDSVDRARALAFPRFDQFIEELSHLQPK**AVPRPRI**GRYQNDQDTN
SFQAKLV

Myomodulin3 (269 aa)

*MGISEASFRFGFVVILLGFSLAHL*DHETTNTARNEANVKAKTDKSVIGPRLQWLLLGKRSANFDSFRPRRQG
RSLDGQDNLGLSSSEIHEALEDFKEEYKR**QPPVPRY**GRDNNDIRFANLDSLRFRLQGLSLNGQDNLGLSSS
EIHEALEDFKEEFKR**QPPVPRY**GRDNNDIRFANFDRQGRSSDGQDSSRLSPNEIQNALDSIFADTKEEFRR**Q**
PPLPRYGRDSSSAR**SFFRPAPRG**GRYKKSLPVGRLMFGDFFSQDGVNQAEDFSL

Pleurin1 (179 aa)

*MNYQNHSLILLVLISSACA***VFYTKSDDNDYPRI**GRR**SFYTRGSDTHYPRI**GRRDATSAQVLIPGLDFADLST
LHKR**GIFTQSAHGSYPRV**GRGSEEASSGEDSLCDQKISLLERLTGLKEEIDEEDGNKEGNSGSDEFRNLFSP
LDATFRKFDVDKDGKLSKKEFLSGFNAVRQNGSGC

Pleurin2 (176 aa)

*MHPPISVLIILALSCAASA***VFFTKASDNDYPRI**GR**SNFFTSGNGNHYPRI**GRRETSRAQILLPGLEFNDLAMA
DKR**GVFTQGPHGSYPRV**GRTSGGRRRGDDYASERKEMSENLEGERVYEIEGNNVGHGGVIDSWHSSVP
LEIMFELFDSDSDGKLSKEEFVSGLSRYPQHRSLY

sCAP1 (133 aa)

*MEMSLSRASLSLTVLALFVFSAEA***MQYLAFPRM**GR**SGYLAFPRM**GRSQTKSETSAEFSNCCGVGLKNEFVV
GGAGKEELRPVCPLNSECCQGLREITDQKPDGTYYSMCVPDFPESSGQSADVLRKLKGLIRK

sCAP2 (133 aa)

*MELTLQRASFSVTVLVLVICSAEA***LNYLAFPRM**GR**SGYLAFPRM**GRGQVKSETGADIGSCCGLGLKSEFLIG
QDGKEELRVLCPANMGCCEGLREVVDEKADGVFYSICVPVQQESNTHESDVLRKAKSLIRK

Figure S1. Prepropeptide sequences of NmU/PRX-encoding genes and their predicted peptide sequences related to NmU/PRX-like peptides from *Deroceras reticulatum*. Signal peptide is represented in italic, the myomodulin (MM) or NmU/PRXamide peptides are in bold and underlined, and the glycine residue (to be amidated) is in green.

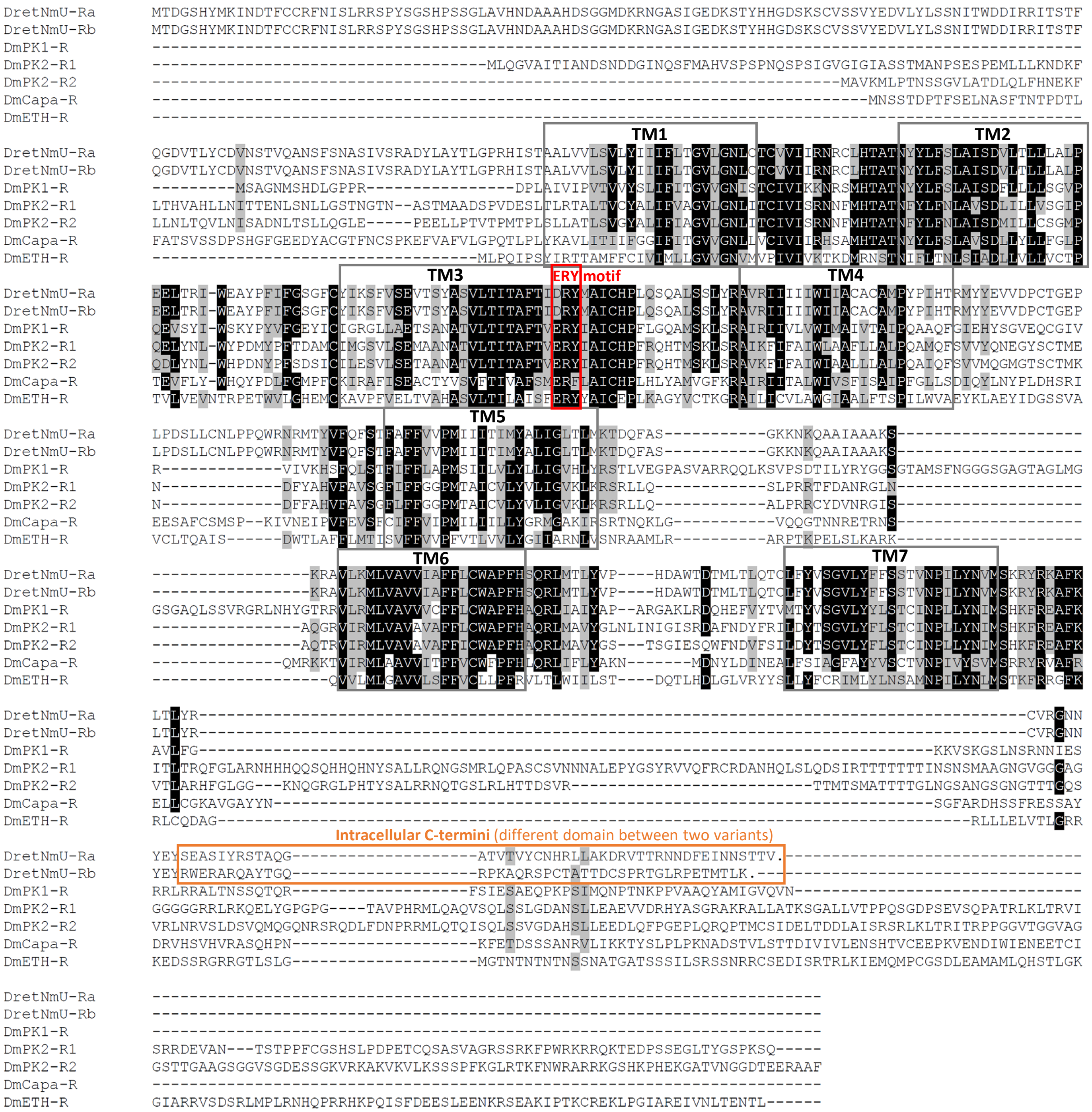

Figure S2. Alignment of deduced amino acid sequences of DretNmU-Ra and DretNmU-Rb from *Deroceras reticulatum* with PRXamide receptors from the fruit fly *Drosophila melanogaster*.

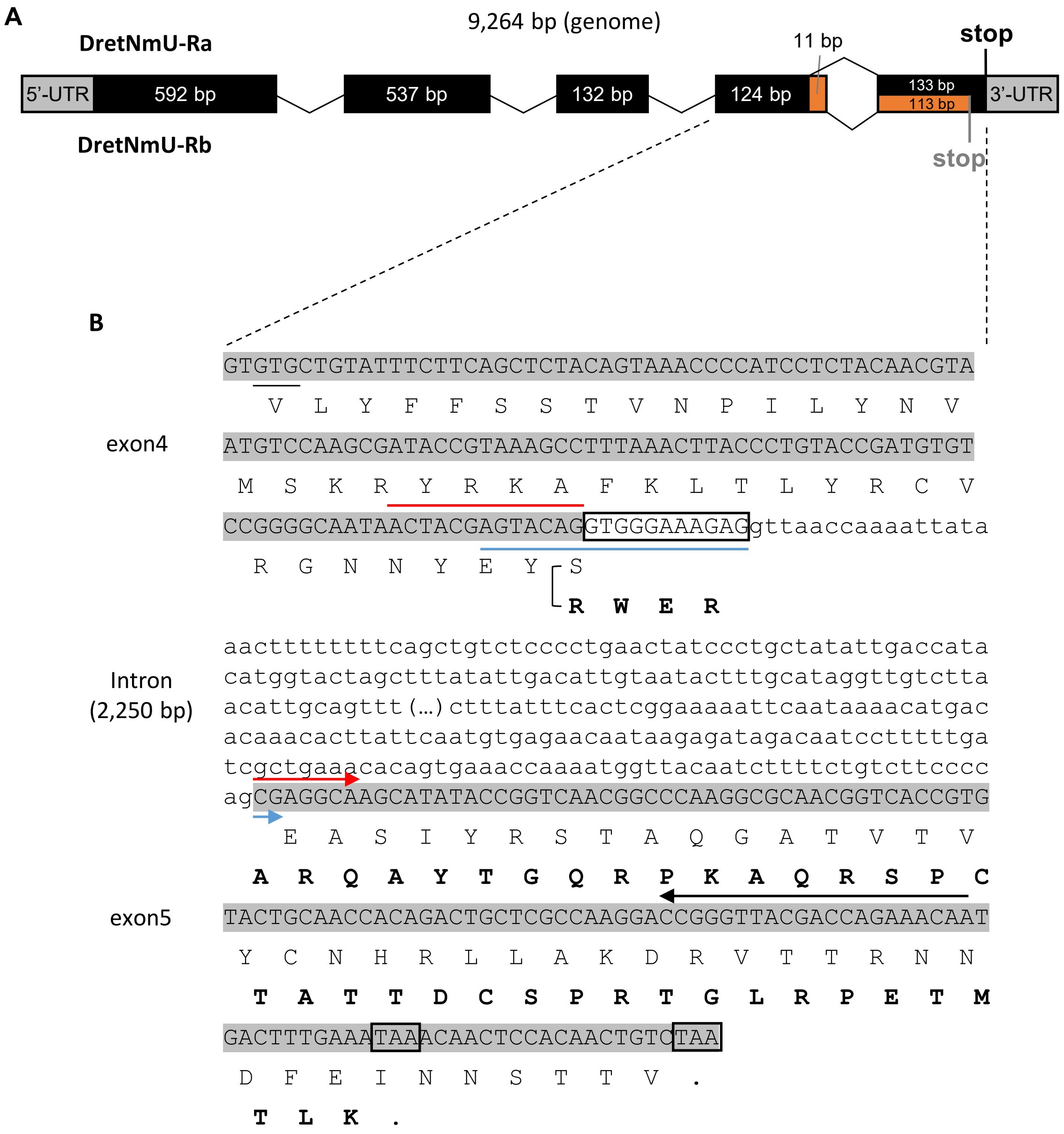

Figure S3. Gene structure of DretNmU-R variants identified from *Deroceras reticulatum*. (A) Exon-intron structure of the DretNmU-R gene. Both variants are composed of five exons (black box), of which exon 4 differs in length. Variant B retains 11 bp of intron sequence (gray box), causing a frame shift. (B) Sequence between exons 4 and 5 (highlighted in gray) including intron (lower case). Deduced amino acid sequences of the corresponding receptor variants DretNmU-Ra and –Rb are below the nucleotide sequences in light and bold letters, respectively. Arrows in red and blue indicate variant-specific forward primers and the arrow in black indicates a reverse primers used for both (See Figure S4). TAA in box refers to stop codon.

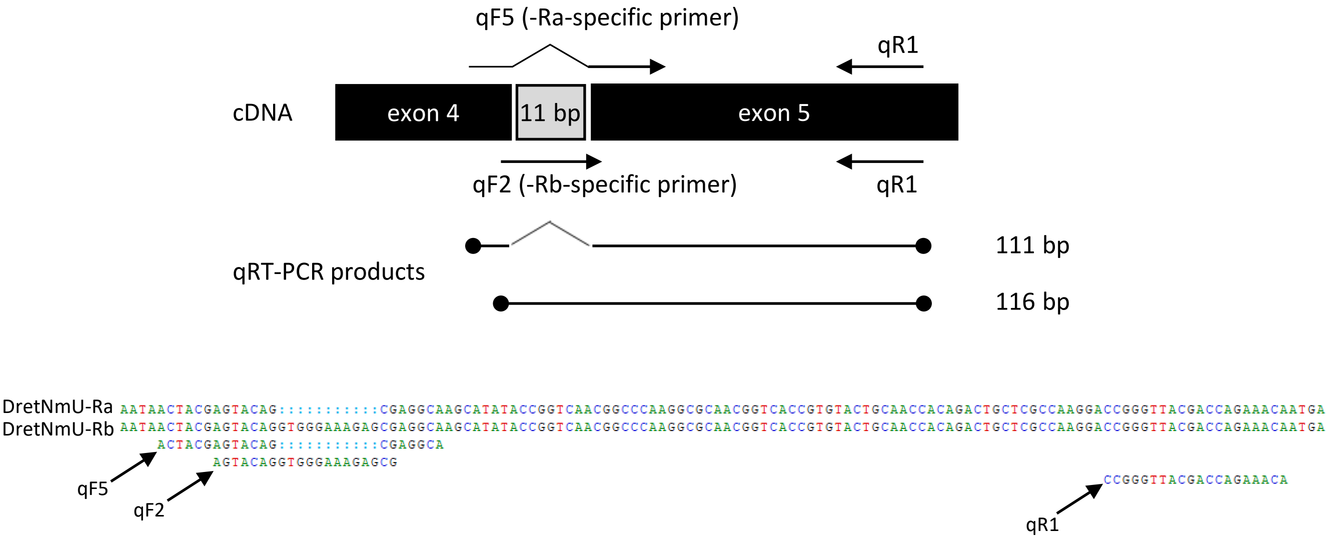

Figure S4. Scheme of the variant-specific qRT-PCR targeting for DretNmU-Ra and DretNmU-Rb, respectively.

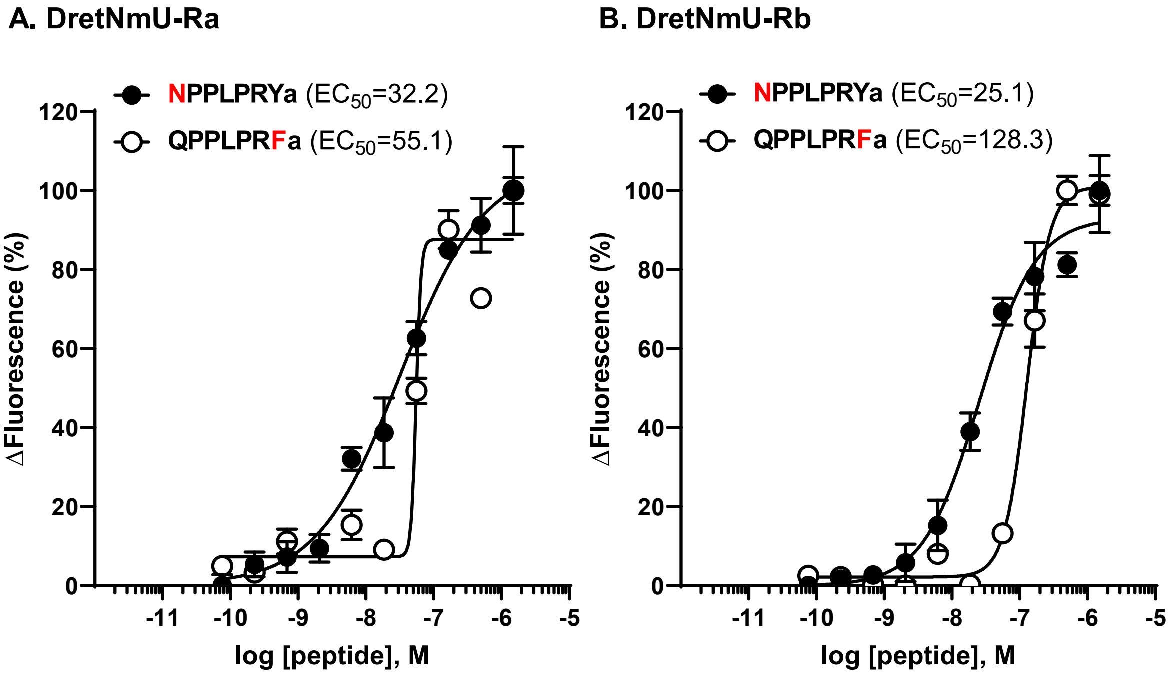

Figure S5. Normalized concentration-responses and EC_{50} values (nM) of the two NmU receptor variants, DretNmU-Ra and DretNmU-Rb, from *Deroceras reticulatum* to the two motified peptides, NPPLPRYa and QPPLPRFa, that elicited high responses in screening experiments (Figure 4). The mean response from four wells receiving the same treatment in the same plate was regarded as one replicate, and four to five replicates from independent assays on different cell plates were analyzed (mean ± SEM).
